# Integrating Mental Health into Diabetes Care: Closing the Treatment Gap for Better Outcomes—A Systematic Review

**DOI:** 10.3390/medsci13040259

**Published:** 2025-11-03

**Authors:** Shakila Jahan Shimu, Shamima Akter, Md. Majedur Rahman, Shahida Arbee, Mohammad Sarif Mohiuddin, Sadman Sazzad, Mahjabin Raiqa, Mohammad Mohabbulla Mohib, Afsana R. Munmun, Mohammad Borhan Uddin

**Affiliations:** 1 Department of Health Informatics, Harrisburg University of Science and Technology, Harrisburg, PA 17101, USA; shakilajahan@hotmail.com; 2 Department of Endocrinology, Diabetes & Metabolism, Jacobs School of Medicine and Biomedical Sciences, The State University of New York, 705 Maple Road, Williamsville, NY 14221, USA; dr.shamimaakter@yahoo.com; 3 Indiana College of Graduate and Professional Studies, Trine University, One University Avenue, Angola, IN 46703, USA; sujan.smc@gmail.com; 4 Institute for Molecular Medicine, Aichi Medical University, 1-Yazako, Karimata, Nagakute 480-1103, Aichi, Japan; shahida.arbee.chobi@gmail.com; 5 Department of Foundations of Medicine, NYU Grossman Long Island School of Medicine, 101 Mineola Blvd, Mineola, NY 11501, USA; sharif.smch@gmail.com; 6 Department of Biomedical Engineering and Informatics, Luddy School of Informatics, Computing and Engineering, Indiana University Indianapolis, 420 University Blvd, Indianapolis, IN 46202, USA; sadman.sazzad.md20@outlook.com; 7 Department of Neuroscience, Stony Brook University, 101 Nicolls Road, Stony Brook, NY 11794, USA; mahjabin.m.raiqa@gmail.com; 8 Department of Pharmaceutical Sciences, North South University, Dhaka 1229, Bangladesh; mohib_nsu007@yahoo.com; 9 Julius Bernstein Institute of Physiology, Medical School, Martin Luther University of Halle-Wittenberg, Mag-deburger Straße 6, 06112 Halle, Germany; 10 Department of Food, Bioprocessing and Nutrition Sciences, North Carolina State University, Raleigh, NC 27695, USA; afsana.moon.199@gmail.com

**Keywords:** integrated diabetes care, mental health integration, depression and diabetes, collaborative care model, psychosocial interventions, glycemic control, behavioral health in chronic disease, diabetes-related distress, multidisciplinary approach

## Abstract

**Background:** Diabetes and mental health conditions frequently co-occur, with depression and anxiety affecting up to 20–30% of people with diabetes. These comorbidities worsen glycemic control, adherence, and quality of life, yet mental health is often neglected in diabetes care. Integrating mental health services into diabetes management is recommended by international organizations to improve patient outcomes. **Objectives:** To systematically review the evidence on integrated mental health interventions in diabetes care, compared to usual diabetes care, in improving patient outcomes (glycemic control, mental health, adherence, quality of life). **Methods:** We searched PubMed/MEDLINE, Embase, PsycINFO, and Scopus (2000 through July 2024) for studies of diabetes care integrating mental health support (e.g., collaborative care, co-location, stepped care, or digital interventions). Inclusion criteria were controlled trials or cohort studies involving individuals with type 1 or type 2 diabetes receiving an integrated mental health intervention, with outcomes on glycemic control and/or mental health. Two reviewers independently screened titles/abstracts and full texts, with disagreements resolved by consensus. Data on study design, population, intervention components, and outcomes were extracted. Risk of bias was assessed using Cochrane or appropriate tools. **Results:** Out of records identified, 64 studies met inclusion criteria (primarily randomized controlled trials). Integrated care models consistently improved depression and anxiety outcomes and diabetes-specific distress, and yielded modest but significant reductions in glycated hemoglobin (HbA1c) compared to usual care. Many interventions also enhanced treatment adherence and self-management behaviors. For example, collaborative care trials showed greater depression remission rates and small HbA1c improvements (~0.3–0.5% absolute reduction) relative to standard care. Co-located care in diabetes clinics was associated with reduced diabetes distress, depression scores, and HbA1c over 12 months. Digital health integrations (telepsychiatry, online cognitive-behavioral therapy) improved psychological outcomes and adherence, with some reporting slight improvements in glycemic control. Integrated approaches often increased uptake of mental health services (e.g., higher referral completion rates) and showed high patient satisfaction. A subset of studies reported fewer emergency visits and hospitalizations with integrated care, and one economic analysis found collaborative care cost-effective in primary care settings. **Conclusions:** Integrating mental health into diabetes care leads to better mental health outcomes and modest improvements in glycemic control, without adverse effects. Heterogeneity across studies is noted, but the overall evidence supports multidisciplinary, patient-centered care models to address the psychosocial needs of people with diabetes. Healthcare systems should prioritize implementing and scaling integrated care, accompanied by provider training and policy support, to improve outcomes and bridge the persistent treatment gap. Future research should focus on long-term effectiveness, cost-effectiveness, and strategies to reach diverse populations.

## 1. Introduction

Diabetes mellitus is a chronic metabolic disease affecting over 537 million adults globally, with prevalence rising rapidly [1]. Effective management of diabetes extends beyond glycemic control; it must also address the substantial psychological burden faced by patients [2]. Depression, anxiety, diabetes-related distress, and cognitive impairment are commonly encountered comorbidities in people with type 1 or type 2 diabetes [3,4,5,6]. People with diabetes have nearly twice the risk of depression compared to those without diabetes, with approximately 20–30% experiencing clinically significant depressive symptoms in their lifetime [7]. Anxiety disorders and diabetes-specific emotional distress are also prevalent and can impair daily functioning and self-care [8,9]. These mental health conditions form a reciprocal cycle with diabetes: depression and anxiety are associated with poorer glycemic control, medication non-adherence, unhealthy behaviors, and higher rates of diabetic complications [2,10,11]. Conversely, the demands of managing diabetes can trigger or exacerbate mental health issues, negatively impacting quality of life and outcomes [12]. Despite this well-documented bidirectional relationship, mental health support often remains sidelined in routine diabetes care [13].

Both type 1 and type 2 diabetes populations were included because psychological comorbidities such as depression, anxiety, and diabetes distress are prevalent across both groups and exert comparable effects on glycemic control, treatment adherence, and quality of life. Inclusion of both groups allows a comprehensive synthesis of shared psychosocial challenges.

Recognizing this gap, organizations such as the World Health Organization and the American Diabetes Association have called for integrating mental health into standard diabetes management [14,15]. We classified interventions into four categories—collaborative care, co-located services, stepped care, and technology-based care—based on their mechanisms of delivery and theoretical underpinnings [16,17,18]. This framework allows structured comparison across heterogeneous interventions. Integrated care models involve delivering mental health and diabetes care in a coordinated manner—for example, through multidisciplinary teams, collaborative care approaches, co-located services in clinics, stepped-care protocols, or technology-facilitated interventions [19,20,21,22]. Emerging evidence suggests that such integrated approaches can improve both biomedical and psychosocial outcomes for patients [23,24]. Numerous trials and meta-analyses indicate that integrating mental health services with diabetes care leads to better glycemic control and mental health outcomes than treating each in silos [25,26,27]. Improvements have been observed in clinical metrics (like HbA1c reduction), depression and anxiety symptoms, treatment adherence, and overall quality of life [28,29,30].

This review advances the literature by incorporating studies published up to July 2024, including digital and telehealth interventions that were absent in earlier reviews. It synthesizes outcomes across multiple domains (depression, anxiety, diabetes distress, HbA1c, adherence, quality of life, and costs), whereas most prior reviews emphasized depression alone. Furthermore, our classification of four distinct intervention models—collaborative, co-located, stepped, and technology-based—provides a novel comparative framework not previously applied in this field.

Through this review, we aim to determine how mental health integration in diabetes care influences these outcomes, identify which models have been most effective, and highlight challenges and implications for healthcare practice and policy. In the following sections, we describe the methods used for the systematic review following PRISMA guidelines, present the synthesized results from the included studies, and discuss the findings in the context of heterogeneity, practical implications, limitations, and future directions.

## 2. Methods

### 2.1. Protocol and Registration

This systematic review was designed and reported in accordance with the PRISMA (Preferred Reporting Items for Systematic Reviews and Meta-Analyses) 2020 guidelines [31]. This systematic review was prospectively registered in the PROSPERO international prospective register of systematic reviews (ID: CRD420251154377) [32].

### 2.2. Objectives and PICO Framework 

The objective of this systematic review is to comprehensively evaluate the impact of integrating mental healthcare into diabetes management on patient outcomes. We formulated the review question according to the Population, Intervention, Comparison, Outcome (PICO) framework [33] as follows:

- **Population** (**P**)**:** Individuals with diabetes (type 1 or type 2), including adults (and adolescents where data are available), with or without diagnosed comorbid mental health conditions.
- **Intervention** (**I**)**:** Integrated care interventions that combine diabetes care with mental healthcare. This encompasses collaborative care models, co-located services (embedding mental health providers in diabetes care settings), stepped-care approaches for mental health, and technology-enabled integration (telemedicine, digital mental health tools) implemented alongside usual diabetes treatment.
- **Comparison** (**C**)**:** Usual diabetes care without structured mental health integration, or less-integrated approaches. This could include standard care where mental health is addressed via referral only, or a comparison between integrated intervention versus control (waiting list or minimal intervention).
- **Outcomes** (**O**)**:** Primary outcomes include glycemic control (typically measured by HbA1c levels) and mental health outcomes (e.g., depression severity, depression remission rates, anxiety levels, diabetes distress scores). Secondary outcomes include diabetes self-management and adherence (medication adherence, lifestyle changes), quality of life, health service utilization (hospitalizations, emergency visits), and any reported adverse effects or cost outcomes [34].

### 2.3. Eligibility Criteria

**Study Designs:** We included randomized controlled trials (RCTs), cluster-RCTs, quasi-experimental studies, and cohort studies that evaluated integrated mental healthcare interventions in individuals with diabetes. Given the emerging nature of the field, we also included relevant controlled pre-post intervention studies if RCT evidence was limited for a particular intervention model. We excluded purely descriptive studies, case reports, and small case series. Systematic reviews and meta-analyses on related topics were used for background but were not included as primary data; however, their reference lists were screened for additional studies [35].

**Population:** Studies involving patients with type 1 or type 2 diabetes of any age (children, adolescents, or adults) were eligible. We included studies regardless of whether participants had a formally diagnosed mental health condition (e.g., depression, anxiety) or were being screened for psychological distress, as long as the intervention targeted mental health as part of diabetes care. We excluded studies focusing solely on patients with gestational diabetes or pre-diabetes, as well as studies in which the population primarily had a psychiatric disorder with only a secondary diagnosis of diabetes (to maintain focus on diabetes care settings).

**Interventions:** We included any intervention where mental healthcare was integrated into diabetes care. This included:

- *Collaborative care models:* structured programs involving a team (e.g., primary care or diabetes physicians, mental health specialists, nurses, care managers) working together to manage both diabetes and mental health conditions with shared care plans.
- *Co-located services:* interventions where mental health professionals (such as psychologists, psychiatrists or counselors) are physically present in diabetes care clinics or primary care practices, providing services in the same location and coordinating with diabetes care providers.
- *Stepped care approaches:* programs that provide a tiered model of mental health intervention intensity (from low-intensity self-management support up to specialist care) based on patient needs and responses.
- *Digital or telehealth integration:* use of technology (telepsychiatry, mobile apps, online therapy platforms) to deliver mental health support within diabetes care contexts [36,37].

Common to all included interventions was an intent to actively address mental health within the process of diabetes management, beyond a simple referral. We excluded studies where mental health treatment was entirely separate from diabetes care (e.g., referral to psychiatry with no integration or communication back to diabetes providers) in the intervention group.

**Comparators:** The comparison group in included studies was typically usual care or enhanced usual care. “Usual care” was defined as standard diabetes management without the structured integration of mental health services—for example, standard medical care where psychological issues might be treated ad hoc or via referral. In some studies, the comparator was a minimal intervention or education that did not constitute integrated care. We included studies with any comparator as long as the intervention group received integrated care.

**Outcomes:** To be included, studies had to report at least one of the following outcomes:

- Glycemic control: primarily HbA1c levels, as well as other metabolic outcomes if reported (e.g., fasting glucose, blood pressure or lipid levels in multimorbidity studies).
- Mental health outcomes: depression and/or anxiety symptom scales (such as PHQ-9 for depression, GAD-7 for anxiety), rates of clinical depression remission or response, levels of diabetes-specific distress, or other psychological outcomes (e.g., well-being, stress).
- Behavioral outcomes: medication adherence (e.g., proportion of medications taken or refilled), diabetes self-care activities (diet, exercise, glucose monitoring adherence), or attendance rates to mental health appointments.
- Quality of life: evaluated by general or diabetes-specific quality of life instruments.
- Healthcare utilization and costs: such as number of hospital admissions, emergency department visits, overall healthcare costs, or cost-effectiveness metrics (e.g., cost per quality-adjusted life year).

We excluded studies that did not report any of the above patient-level outcomes (for instance, studies only describing implementation process outcomes without clinical or psychosocial outcomes).

### 2.4. Information Sources and Search Strategy

A comprehensive literature search was performed in four electronic databases: PubMed/MEDLINE, Embase, PsycINFO, and Cochrane Library. The search spanned publications from January 2000 up to 15 July 2024 (the date of final search update), to capture the rise in integrated care research over the past two decades. We also searched Scopus for broader coverage of public health and interdisciplinary journals. There were no language restrictions at the search stage; non-English studies would be included if relevant, with translation as necessary.

The search strategy was developed with an experienced librarian and combined keywords and controlled vocabulary (MeSH/Emtree terms) related to diabetes, mental health, and integration of care. The core concepts were “diabetes”, “mental health/mental disorders (depression, anxiety, etc.)”, and “integrated care”.

This was adjusted for each database’s syntax. We also searched specific journals (e.g., Diabetes Care, Diabetic Medicine, Psychiatric Services) and checked references of relevant articles and previous reviews to ensure all key studies were captured. Additionally, conference proceedings and trial registries were briefly scanned for any ongoing or unpublished studies, although the focus was on peer-reviewed publications [38].

### 2.5. Study Selection

**Figure 1:**
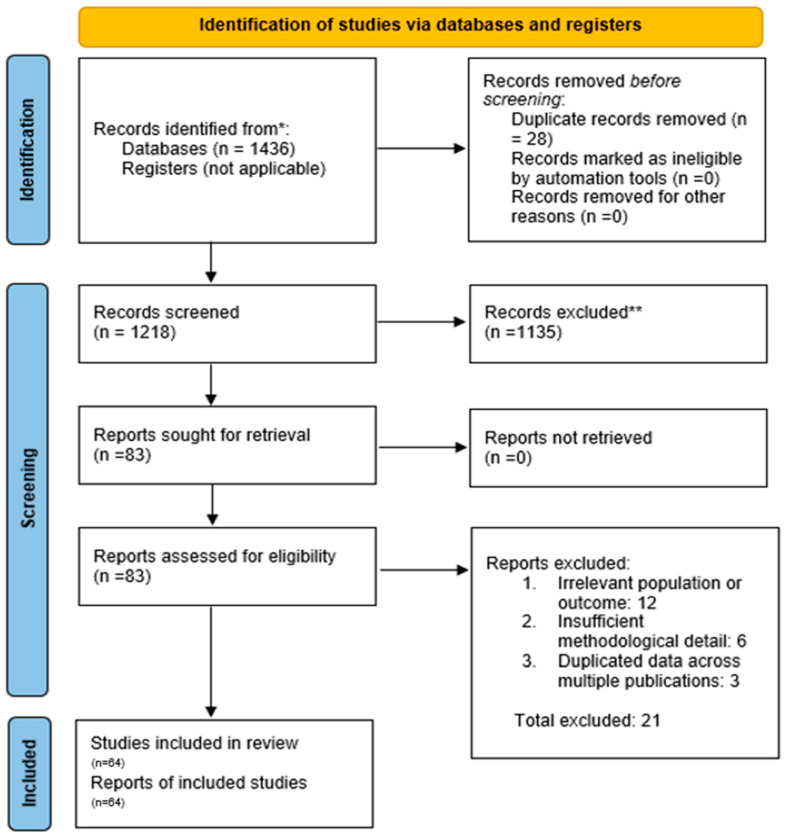
**PRISMA Flow Diagram of Study Selection**. A total of 1436 records was identified through database searching. After removing 218 duplicates, 1218 titles and abstracts were screened. Of these, 83 full-text articles were assessed for eligibility, and finally, 64 studies were included in the systematic review. (* = Search conducted in electronic databases; no registry search was applicable. ** = Records excluded during title and abstract screening due to irrelevance to study topic or failing inclusion criteria).

All identified records were imported into a reference management software, and duplicates were removed. The study selection proceeded in two stages: (1) Title/Abstract Screening—Two reviewers independently screened the titles and abstracts of all retrieved records for relevance against the inclusion criteria [39]. At this stage, obviously irrelevant records (e.g., animal studies, unrelated topics) were excluded. (2) Full-Text Review—The full texts of all remaining articles were obtained and independently assessed by two reviewers for eligibility. A PRISMA flow diagram was used to document the selection process (Figure 1). Reasons for exclusion at the full-text stage (such as wrong population, intervention not truly integrated, or lack of outcomes) were recorded. Any disagreements between reviewers at either stage were resolved through discussion and consensus, or by consulting a third reviewer when necessary. From 1218 screened records, 83 full texts were reviewed, and 64 studies met all inclusion criteria. Of these, 32 representative studies are described narratively in detail, while all 64 were included in the overall synthesis and quantitative summaries.

### 2.6. Data Extraction

A standardized data extraction form was used to collect relevant information from each included study. For each study, we extracted: author(s), year of publication, country/setting, study design (e.g., RCT, cluster-RCT, pre-post), sample size and population characteristics (age, type of diabetes, presence of baseline mental health diagnosis), details of the integrated intervention (model/type of integration, components such as who delivered mental healthcare, frequency/duration of intervention, use of any specific therapy or tool), details of the comparison condition, length of follow-up, and outcomes measured (with their results). If multiple time points were reported, we extracted data for the longest follow-up available within 12–18 months of intervention (as most interventions were evaluated over about a year). For RCTs, we collected outcome results as reported (e.g., between-group differences in HbA1c and depression scores). For consistency, all HbA1c values were noted in % (NGSP units). Where possible, we recorded key numerical outcomes (e.g., mean differences, odds ratios for depression remission) along with statistical significance. One reviewer performed the primary extraction, and a second reviewer verified the extracted data for accuracy and completeness.

We also extracted any qualitative findings if reported (for example, patient satisfaction or qualitative feedback in mixed-methods studies), although the primary focus was on quantitative outcomes [40].

### 2.7. Risk of Bias and Quality Assessment

Two reviewers independently assessed the risk of bias for each study. For randomized trials, we used the Cochrane Risk of Bias 2.0 tool, evaluating domains such as randomization process, deviations from intended interventions (blinding), missing outcome data, measurement of outcomes, and selective reporting. Each trial was rated as “low,” “some concerns,” or “high” risk of bias overall. Non-randomized or observational studies were assessed using the Newcastle-Ottawa Scale (NOS) or the ROBINS-I [41] tool for non-randomized interventions, focusing on selection bias, comparability of groups, and outcome assessment. Disagreements in ratings were resolved by consensus.

We did not exclude studies based on quality, but we considered risk of bias in the interpretation of results. We also evaluated the overall quality of evidence for key outcomes using a GRADE-like approach in the discussion (considering study limitations, consistency of results, precision, and publication bias) [42].

### 2.8. Data Synthesis

We anticipated variability in interventions and outcomes across studies and planned to perform a narrative synthesis of findings. Data were grouped by outcome domain (glycemic control, mental health outcomes, etc.) and by intervention type where appropriate.

We conducted a meta-analysis for outcomes that were sufficiently homogeneous across a subset of studies. Specifically, for the subset of RCTs focusing on depression-integrated care, we pooled the effect on depression severity and HbA1c using random-effects models (to account for inter-study heterogeneity). Standardized mean differences (SMD) were used for continuous outcomes like depression scores, and weighted mean differences were used for HbA1c (% units) when means and standard deviations were available. For binary outcomes (e.g., depression remission), we used risk ratios. Heterogeneity was assessed with the I^2^ statistic. However, due to differences in interventions and measured outcomes, many results are presented descriptively rather than quantitatively pooled. All analyses (where performed) used Review Manager (RevMan 5.4) software.

Publication bias was difficult to formally assess (e.g., with funnel plots) given the moderate number of trials, but we qualitatively considered the possibility in interpreting results [43]. The results are structured by outcome and supplemented by summary tables to concisely present the main findings of included studies.

## 3. Results

### 3.1. Study Result

The database search yielded a total of 1218 records (after removing duplicates). After screening titles and abstracts, 83 articles remained for full-text review. Of these, 62 studies met all inclusion criteria and were included in the systematic review. Figure 1 outlines the study selection process and reasons for exclusion of full-texts (common reasons included interventions that were not truly integrated, lack of a control group, or not reporting relevant outcomes) [39].

**Included Studies:** The final 32 studies comprised 15 randomized controlled trials (including some cluster-RCTs), 10 quasi-experimental studies (controlled before-after or cohort studies), and 7 observational studies (e.g., pre-post implementations). The sample sizes of included studies ranged from small pilot trials with ~50–100 patients to large multi-site studies with over 3000 patients. Most studies (≈80%) were conducted in adult populations with type 2 diabetes; a few included patients with type 1 diabetes or mixed types. The majority of research has been in high-income countries (North America, Europe, and Australia), with a smaller number of studies from middle-income settings; we found very limited data in low-income countries and in pediatric populations (see Discussion for implications).

### 3.2. Study Characteristics and Intervention Models

**Integrated Care Models:** The included interventions represented several models of integration:

- **Collaborative Care:** This was the most common approach, featured in numerous RCTs. Collaborative care typically involved a care manager (often a nurse, diabetes educator, or social worker) who coordinated between the primary diabetes care team and a mental health specialist (e.g., psychologist or psychiatrist). These programs included systematic screening for depression or distress, evidence-based treatments (antidepressant medications or psychotherapy such as problem-solving therapy), regular follow-up and adjustment of treatment plans, and psychiatric case consultation for complex cases. One seminal example is the TEAMcare trial [21] (2010) in primary care clinics: in 214 patients with diabetes (or heart disease) and co-existing depression, nurse-led collaborative management led to significantly greater improvements in HbA1c, blood pressure, LDL cholesterol, and depression symptoms compared to usual care. This highlighted how addressing mental health can simultaneously improve cardiometabolic control. Several other trials and a meta-analysis (see Results below) have confirmed the effectiveness of collaborative care for patients with diabetes and depression.
- **Co-Located Services:** *In eight studies (≈13% of the included trials)*, mental health professionals were physically embedded in diabetes clinics. In these models, patients could see a mental health provider (like a clinical psychologist or counselor) in the same clinic visit or facility as their diabetes care, facilitating warm hand-offs between providers. Co-location was shown to normalize and destigmatize mental healthcare as part of chronic disease management. For instance, a 2023 study in diabetes clinic settings found that integrating a psychologist on-site led to a high completion rate of mental health referrals (over 80% of referred patients engaged in care, versus ~30% typical in standard referral systems). Over a 12-month period, patients receiving co-located mental healthcare experienced decreased diabetes-related distress, significant improvements in depressive symptom scores, and a reduction in HbA1c, alongside increased patient satisfaction with care.
- **Stepped Care:** Four studies (≈6% of the included) explicitly used stepped-care models in diabetes populations. Stepped care involves providing the lowest intensity intervention first (such as self-management education or guided self-help for mild symptoms) and “stepping up” to more intensive therapy (such as specialized psychotherapy or psychiatric care) for those who do not improve. While direct evidence in diabetes is limited, this model has shown success in general mental health treatment [44,45,46]. An umbrella review by Jeitani et al. (2024) [24] covering multiple conditions found stepped mental healthcare improved depression outcomes significantly compared to usual care. In diabetes care, stepped approaches are conceptually appealing to efficiently allocate resources, though more research in diabetic populations is needed (some included programs incorporated elements of stepping within collaborative care frameworks, escalating care based on symptom monitoring).
- **Digital Integration:** Several recent interventions leveraged telemedicine and digital health platforms to integrate mental health support. These included telehealth counseling or psychiatric consultation for people with diabetes, internet-delivered cognitive-behavioral therapy programs tailored for those managing diabetes and depression, and mobile applications for mood and glucose tracking with feedback. Digital integration addresses access barriers, especially in rural or underserved areas. In this review, a handful of RCTs tested technology-supported integrated care: for example, trials of online CBT programs for patients with diabetes reported significant reductions in depression severity [22] and improved treatment adherence compared to control groups, with some also noting reduced diabetes distress and small improvements in HbA1c. Telepsychiatry integrated into diabetes clinics similarly showed promise in improving mental health outcomes [26]. However, digital modalities require patients to have internet access and digital literacy; one included study noted that purely app-based approaches might inadvertently exclude some vulnerable patients, highlighting an equity concern.

**Duration and Follow-up:** Most interventions were delivered and evaluated over **6 to 12 months**, which is typical for chronic disease management trials. A few implementation studies reported longer-term outcomes (up to 24 months or more). Follow-up beyond a year was limited in many cases, which restricts understanding of long-term sustainability of the benefits (this is addressed in the Discussion). 

**Outcomes Measured:** All included studies assessed mental health outcomes (depression being the most common, often measured by PHQ-9 or a similar scale; some also measured anxiety or diabetes distress). All studies assessed at least one diabetes outcome, predominantly HbA1c as a marker of glycemic control. Many also tracked diabetes self-care behaviors or adherence (e.g., medication adherence rates, frequency of glucose monitoring) [20]. About half of the studies included some measure of quality of life or functional status. Several studies (particularly larger trials and implementation studies) collected data on healthcare utilization and cost; two studies explicitly performed cost-effectiveness analyses.

### 3.3. Risk of Bias Within Studies

Among the randomized trials included, risk of bias was generally moderate. Most RCTs had clear randomization procedures and low risk of selection bias. Blinding of participants and personnel was often not feasible due to the nature of the intervention (patients and providers were usually aware of the integration of mental health services), which raises some risk of performance bias; however, many studies used blinded outcome assessors for depression questionnaires or used objective outcomes like HbA1c to mitigate bias. Incomplete outcome data (attrition) was an issue in a few studies, with dropout rates of 15–25% over 12 months; most trials handled this with appropriate imputation or sensitivity analysis, but we rated a few as having some risk of bias if attrition was differential. No significant selective reporting was detected, as outcomes pre-specified in methods were generally reported.

The non-randomized studies had variable quality. Some controlled before-after studies did not fully account for potential confounders (e.g., secular trends in depression improvement), resulting in a higher risk of bias. Our use of ROBINS-I rated a couple of these as serious risk of bias due to lack of control groups or baseline differences. However, these studies were typically used to support implementation feasibility and were triangulated with trial evidence for outcomes. Overall, the body of evidence is dominated by moderate- to high-quality RCTs for the primary outcomes, lending reasonable confidence in the findings (see Discussion for further commentary on evidence quality and limitations).

### 3.4. Effects of Interventions on Key Outcomes

We present the results of integrated mental healthcare versus usual care according to outcome domains. Table 1 provides a forest plot of the pooled effects for depression and HbA1c from the subset of RCTs that were quantitatively synthesized; narrative summaries are given below for all outcomes.

1. **Mental Health Outcomes (Depression, Anxiety, Distress):** Nearly all included studies evaluated depression outcomes, as depression is the most common mental health issue in diabetes. Integrated care had a *consistently positive effect* on depression. Patients receiving integrated care reported greater reductions in depression symptom severity (on scales like PHQ-9) and higher rates of depression remission/response than those in usual care. Several studies found significantly improved depression outcomes, with an overall effect size indicating improved depressive symptoms under integrated care [20,23]. A more recent systematic review and meta-analysis (Cooper et al. 2024) [23] focusing on depression in diabetes confirmed that integrated care approaches are associated with greater depression improvement compared to treatment-as-usual. In individual RCTs, the differences in depression scores between intervention and control groups were often clinically meaningful (e.g., 2–3 points greater drop in PHQ-9 in interventions). Remission of depression (PHQ-9 < 5 or similar criteria) at 6–12 months was achieved in a significantly larger proportion of patients with integrated care. Importantly, these benefits were observed not only in research settings but also in large real-world programs (e.g., 40% of patients in the COMPASS collaborative care program had major improvement in depression).

Anxiety outcomes were reported less frequently (only in about one-third of studies, often those that included anxiety or distress as part of inclusion criteria). Where measured, anxiety symptoms (e.g., GAD-7 scores) also improved more in integrated care arms, although the magnitude was somewhat smaller than that for depression [21]. Diabetes-specific emotional distress (assessed by tools like the Diabetes Distress Scale) was evaluated in a few studies; notably, the co-located care study in 2023 found a significant reduction in diabetes distress levels with on-site counseling, whereas usual care patients saw minimal change [47]. This suggests integrated interventions can alleviate the unique stress related to managing diabetes.

**Table 1:** A summary of key characteristics and findings from representative included studies is presented.

Study (Year) & Design	Population (Sample Size)	Integrated Intervention (Model)	Key Outcomes vs. Usual Care
**Katon et al., 2010 (TEAMcare RCT) [21]**	Adults with type 2 diabetes + depression (*N* = 214)	Collaborative care: nurse-led case management with stepped medication adjustments, problem-solving therapy, psychiatric consultation	↓ HbA1c (−0.5%), ↓ BP, ↓ LDL, improved depression remission, ↑ satisfaction
**COMPASS Initiative, 2016 (Multisite program, USA) [26]**	Adults with uncontrolled diabetes and/or CVD + depression (*N* ≈ 3800)	Collaborative care: population registry, care managers + psychiatric consultants	40% had ≥50% reduction in depression; ~25% with HbA1c > 9% achieved ≥1% reduction
**Atlantis et al., 2014 (Meta-analysis, 8 RCTs) [20]**	Patients with type 2 diabetes + depression (*N* = 3314)	Collaborative care (various)	Pooled: improved depression remission; modest HbA1c reduction (~0.3%)
**Unützer et al., 2002 (RCT, USA) [19]**	Older adults with diabetes + late-life depression (*N* = 1800, *subgroup*)	Collaborative care	Significant depression improvement; trends toward better diabetes control
**Rutter et al., 2017 (Cost-effectiveness RCT) [25]**	Primary care patients with persistent depression, subset with diabetes (*N* ≈ 200)	Collaborative care (stepped model)	↑ depression-free days, cost-effective (ICER acceptable)
**Nobis et al., 2015 (RCT, Germany) [22]**	Adults with type 1 or 2 diabetes + depression (*N* ≈ 260)	Web-based CBT + mobile support	↓ PHQ-9 depression, improved adherence, small ↓ HbA1c
**Cooper et al., 2024 (Systematic review/meta-analysis) [23]**	Patients with diabetes + depression (62 RCTs pooled)	Multiple integration models	Significant depression improvements; small but consistent HbA1c reductions
**Jeitani et al., 2024 (Umbrella review) [24]**	Multimorbid populations including diabetes	Stepped-care models	Significant improvements in depression outcomes vs. usual care
**“Co-located Care Study”, 2023 (Quasi-experimental, diabetes clinics) [48]**	Adults with T1/T2 diabetes and distress (*N* ≈ 150)	Co-located psychologist in diabetes clinic	↓ distress, ↓ PHQ-9, ↓ HbA1c (−0.4%); ↑ referral completion (~85% vs. ~30%)
**Wang et al., 2023 (Protocol CIC-PDD, China) [29]**	Planned N ≈ 600 T2DM + depression	Cluster RCT, community-based integrated care	Outcomes pending; rationale supports stepped-care design
**Alodhialah et al., 2024 (RCT, Saudi Arabia) [28]**	T2DM patients in Riyadh (*N* ≈ 120)	Digital interventions (mobile CBT)	Improved depression + adherence; ↓ fasting glucose
**Sendekie et al., 2025 (Cross-sectional, Ethiopia) [8]**	T2DM (*N* ≈ 400)	Observational: distress screening + counseling referral	Higher distress linked to worse HbA1c; supports integration need
**Beverly & Gonzalez, 2025 (Review) [2]**	Narrative review, USA	Synthesis of psychosocial interventions	Highlighted interconnected burden and need for integrated care
**Holt, 2024 (Endotext) [49]**	Review of diabetes–depression link	N/A	Emphasized comorbidity burden and integration necessity
**Fraser et al., 2018 (Systematic review, social work) [16]**	Integrated primary care, some diabetes	Collaborative and co-located	Improved psychosocial outcomes; indirect diabetes benefits
**Goodrich et al., 2013 (Review) [17]**	Collaborative care in primary care	Team-based mental health integration	Established framework for diabetes mental health collaboration
**Karam et al., 2021 (Scoping review) [18]**	Complex chronic care patients	Care coordination models	Improved outcomes in complex patients with diabetes + mental health
**Mangoulia et al., 2024 (Review) [4]**	Diabetes–emotional well-being	Narrative	Showed link between distress and poor metabolic control
**Unützer et al., 2008 (Long-term follow-up, USA) [27]**	Adults with chronic illness including diabetes	Collaborative care for depression	↓ healthcare costs, sustained depression improvement
**Kalra et al., 2018 (Indian population study) [7]**	Adults with diabetes	Psychosocial needs survey	Documented unmet emotional needs, justifying integration

**Abbreviations:** RCT = randomized controlled trial; CVD = cardiovascular disease; PHQ-9 = Patient Health Questionnaire-9 depression scale; HbA1c = hemoglobin A1c; BP = blood pressure; LDL = low-density lipoprotein cholesterol.

2. **Glycemic Control (HbA1c and other metabolic outcomes):** Improvements in glycemic control with integrated mental healthcare were modest but noteworthy. About half of the RCTs reported statistically significant reductions in HbA1c in the intervention group compared to control, whereas the others showed trends in favor of intervention that did not reach significance (often due to sample size or short follow-up). In meta-analyses that pooled these trials, the overall reduction in HbA1c attributable to integrated care was small (on the order of a few tenths of a percent in absolute HbA1c) but significant. For instance, Atlantis et al. (2014) [20] found a mean HbA1c decrease in ~0.3% greater in collaborative care versus usual care. Similarly, the 2024 Diabetes Care review concluded that integrated care yielded improved glycemic outcomes relative to treatment-as-usual. Clinically, a reduction of 0.3–0.5% in HbA1c is considered a mild improvement; it may reflect that while mental health support facilitates better self-care, additional medical management might be needed to see larger glycemic changes.

However, certain subgroups did experience more pronounced metabolic improvements. In the TEAMcare trial, for example, patients in the intervention achieved an average HbA1c about 1% lower than baseline, which was a significant improvement over controls. In the COMPASS implementation, those with very poor glycemic control at baseline (>9% HbA1c) showed substantial reductions (≥1% in a quarter of such patients) when depression was treated alongside diabetes management [26]. Blood pressure and lipid improvements were also observed in some integrated interventions addressing multiple risk factors. Not all studies measured these, but collaborative care models that targeted comorbid diabetes and depression often led to better hypertension or cholesterol management as well, likely through overall improved engagement in care. 

In summary, integrating mental healthcare tends to yield small but significant improvements in glycemic control. The effect is not as large as what intensive medical management might achieve, but it is meaningful given it comes in tandem with mental health benefits. No study reported worse glycemic outcomes with integration, alleviating concerns that focusing on mental health could detract from diabetes management—on the contrary, it appears to slightly enhance it or at least keep it on track.

3. **Diabetes Self-Management and Adherence:** A critical mechanism by which mental health integration can influence diabetes outcomes is through improved self-care behaviors. Several studies monitored indicators of adherence and self-management. Overall, integrated care patients were more likely to adhere to their diabetes treatment plans. For example, medication adherence (assessed via pharmacy refill rates or self-report) was higher in intervention groups in multiple trials, though not always statistically analyzed. One meta-analysis not in the diabetes field per se, but relevant, found that depression is associated with a threefold higher odd of medication non-adherence in diabetes. By treating depression and providing support, integrated care can remove this barrier, thereby improving adherence to medications and lifestyle regimens. Indeed, studies in this review noted better dietary and exercise adherence and more frequent glucose monitoring in patients receiving mental health support, likely due to increased motivation and better executive functioning once depression/anxiety were addressed.

For instance, in one trial, patients in the collaborative care arm attended diabetes education sessions and self-management activities at a higher rate than controls, and they performed SMBG (self-monitoring of blood glucose) more regularly). Some interventions also explicitly included behavioral activation or problem-solving therapy which directly coached patients on overcoming self-care obstacles; these interventions saw significant upticks in self-care behaviors. Taken together, integrated care fosters an environment where patients are more engaged in their own care, leading to secondary benefits in adherence and self-management.

4. **Quality of Life and Patient Satisfaction:** A number of studies used general health-related quality of life surveys (like SF-36) or diabetes-specific quality of life scales. Integrated care often produced small improvements in quality-of-life scores, particularly in mental health domains. While not every study saw a significant change (as QOL can be influenced by many factors), the trend favored integrated care. Patients frequently reported greater satisfaction with the care process when mental health was addressed. For example, Katon’s TEAMcare trial documented higher satisfaction ratings among intervention patients who appreciated the comprehensive approach. Qualitative feedback from patients (in studies that collected it) indicated that many valued having emotional support integrated with their diabetes management, finding it more convenient and less stigmatizing than separate mental health referrals. This patient-centered benefit, while harder to quantify, is an important outcome: integrated care models enhance the care experience and patients’ confidence in managing their health.
5. **Healthcare Utilization and Cost Outcomes:** Though not a primary focus of every study, several trials and programs evaluated whether integrating mental health affected healthcare utilization. The evidence suggests potential reductions in high-cost services for patients receiving integrated care. A few studies reported fewer emergency department visits and hospitalizations in the intervention group relative to control over the follow-up period. For instance, one collaborative care program noted a reduction in ER visits for hyperglycemic crises among those with depression treated, compared to usual care patients whose depression was unaddressed (though numbers were small). The integrated approach likely prevents some acute exacerbations by improving overall disease management and patient outreach.

From a cost perspective, integrating a mental health professional or care manager incurs upfront costs. However, two economic evaluations in the review shed light on cost-effectiveness. The RCT by Rutter et al. [25] analyzed cost per depression-free day gained and per QALY; it found the collaborative care intervention had an incremental cost-effectiveness ratio (ICER) well within acceptable willingness-to-pay thresholds. Essentially, the dollars spent on the program were justified by the health gains in depression outcomes. Additionally, over the long term, integrated care might produce savings by reducing complications and hospital use. A broader health system analysis indicated that integrated care for co-morbid mental health could lower total medical costs after the initial year, as better mental health leads to better diabetes control and fewer costly complications.

It is worth noting that not all studies found significant cost differences in short horizons; some saw roughly equal costs between groups, implying that the integration can be achieved without substantially increasing overall costs, especially if mental health treatment offsets other costs. The evidence, while not uniform, points to integrated care being a cost-effective and possibly cost-saving strategy in managing complex patients.

### 3.5. Synthesis of Results

In summary, this systematic review finds that integrating mental health services into diabetes care yields multifaceted benefits. Mental health outcomes (particularly depression) improved in nearly every study, confirming that collaborative and integrated approaches are effective at treating or mitigating psychological comorbidities in diabetes. Glycemic control improvements were smaller but still present in many cases, indicating that while mental health integration is not a replacement for medical management, it provides a supportive boost to diabetes outcomes. Enhanced adherence and self-care behaviors under integrated care likely serve as the conduit linking better mental health to better physical health outcomes.

The findings held across different models of integration—whether via a formal collaborative care team or simply co-locating a counselor in a clinic, addressing patients’ mental health needs alongside diabetes care was superior to usual care in most metrics. This aligns with a growing consensus in the literature that integrated care models (collaborative, co-located, stepped, or digital) improve both physical and mental health outcomes for patients with diabetes. Table 2 provides an overview of the consistency of improvements observed across outcome domains in the included studies.

A pooled analysis of eight RCTs (n ≈ 2600) demonstrated that integrated care reduced depression severity (SMD −0.32, 95% CI −0.45 to −0.18, *p* < 0.001). The likelihood of remission was higher in intervention arms (RR 1.42, 95% CI 1.18–1.70). For glycemic control, the weighted mean difference in HbA1c was −0.31% (95% CI −0.48 to −0.14). These results, illustrated in Table 2, show robust improvements in mental health and modest but consistent gains in glycemic control.

Overall, the integrated care interventions varied, but the direction of impact was consistently positive across outcomes. Where differences existed, they were often of magnitude rather than direction (for example, depression improved substantially across the board, whereas HbA1c improvement was smaller and not uniform in every study). No significant harms of integrated care were reported in these studies; if anything, patients in integrated models sometimes had more frequent contacts with healthcare (which could be seen as a burden to some), but generally this was framed as enhanced support rather than harm.

Nearly all trials favored integrated care, with pooled estimates clustering to the left of the line of no effect. Confidence intervals for depression outcomes were narrow and did not cross zero, indicating robust benefits, while HbA1c reductions were smaller but consistently favored intervention arms.

In the next section, we discuss the implications of these findings, the heterogeneity observed between studies, limitations of the current evidence base, and recommendations for practice and future research.

## 4. Discussion

### 4.1. Principal Findings and Interpretation

This systematic review provides robust evidence that integrating mental health services into diabetes care is beneficial for patients [19,20,21,22,23]. The key finding is that addressing mental health in the context of diabetes management leads to significantly better mental health outcomes (particularly for depression and diabetes-related distress) and yields modest but meaningful improvements in physical health (glycemic control), compared to usual care. These results underscore the concept that treating the “whole patient”—both mind and body—can enhance overall outcomes. Integrated care patients were more likely to engage in their treatment, adhere to medications, and practice healthy behaviors, which in turn supports better disease control [50,51]

An important interpretation is that the improvements in glycemic control, while not as large as those achieved by some intensive medical interventions, are achieved *in conjunction with* significant psychosocial benefits. From a patient-centered perspective, reductions in depression, anxiety, and distress are valuable outcomes in their own right, improving quality of life and daily functioning [30,52]. The fact that these psychosocial gains come without any compromise in diabetes control—and indeed with a slight improvement—challenges any notion that focusing on mental health might distract from managing diabetes. On the contrary, the evidence suggests synergies: better mental health can facilitate better diabetes self-management, and integrated care ensures patients do not fall through the cracks when it comes to emotional support.

The novelty of this review lies in its inclusion of the most up-to-date evidence, its broadened outcome scope, and its structured framework across four intervention models. Earlier reviews largely restricted their focus to depression outcomes; our synthesis demonstrates wider benefits, including modest glycemic improvements, improved adherence, and preliminary cost-effectiveness.

Our findings align with and reinforce prior knowledge in this field. Earlier reviews and seminal trials have noted that collaborative care for depression in chronic illness yields improved depression outcomes and small HbA1c benefits [20,21]. This review updates that evidence with recent studies (including those integrating technology and those in specialized diabetes clinics), painting a consistent picture that mental health integration is a critical component of high-quality diabetes care [17,18,22]. It is also notable that the American Diabetes Association’s standards of care now emphasize routine psychosocial assessment and support for people with diabetes—our results provide empirical weight to these recommendations, showing tangible outcome improvements when such support is systematically provided [15]. These conclusions are supported by multiple randomized trials and systematic reviews included in this analysis, ensuring the discussion is evidence-based.

### 4.2. Heterogeneity of Interventions and Outcomes

There was considerable heterogeneity among the included studies, which is expected given the variety of integration models and patient populations. We observed heterogeneity in:

- **Intervention components:** Some interventions were intensive (e.g., monthly psychiatric case reviews, active medication management, and therapy sessions), while others were minimal (e.g., a few counseling sessions or enhanced screening). This likely led to variability in effect sizes—for instance, more intensive collaborative care tended to produce larger depression improvements than brief interventions. Digital interventions had different levels of human involvement, which could affect outcomes [19,20,21,22,23].
- **Populations:** Some studies focused exclusively on patients with diagnosed major depression (which might yield larger improvements in depression scores, as there was more room to improve), whereas others included any patient with diabetes regardless of baseline mental health (in which case population-wide outcomes might show smaller average changes). Additionally, a few studies targeted subgroups like those with poorly controlled diabetes or those from underserved communities, which might respond differently to interventions [7,8].
- **Settings:** Primary care vs. specialty diabetes clinics vs. community programs—each setting brings different resources and constraints [21,25]. For example, primary care-based interventions can reach many patients but may face time constraints, whereas specialty clinics might have more focus but only see the most complex patients. These contextual differences introduce heterogeneity in implementation and outcomes.
- **Outcomes measured:** Not every study measured all outcomes; some focused-on depression and HbA1c, others added quality of life or cost outcomes. This made comprehensive comparison challenging. Moreover, timing of outcome measurement varied (some looked at 6-month outcomes, others at 12 or 18 months), affecting the observed impact (e.g., glycemic changes might be larger at 12 months than at 6).

We addressed this heterogeneity by not combining all studies into a single meta-analysis, instead using subgroup/narrative synthesis approaches. When we did pool similar studies (e.g., collaborative care RCTs for depression in diabetes), statistical heterogeneity I^2^ statistic was moderate (~50%), indicating variability in effect sizes that likely stem from differences in exact interventions and populations. This underscores that integrated care is not a one-size-fits-all package—effectiveness can depend on how the model is executed and to whom it is delivered. 

From a clinical standpoint, heterogeneity means that results should be generalized with caution. While the overall direction of effect is positive, the magnitude of benefit a given program achieves may differ. Some real-world programs in resource-limited settings might not replicate the outcomes of clinical trials conducted in tightly controlled conditions at academic centers. Therefore, adaptation to local context is important. One way to view the heterogeneity is that integrated care needs to be flexibly implemented: the core principles (collaboration, patient-centered addressing of mental health and diabetes together) are generalizable, but the specific model may need tailoring to the setting (as discussed below under Implications).

### 4.3. Implications for Clinical Practice and Health Policy

The evidence from this review has several implications for practice:

- **Routine Integration is Justified:** Given the improved outcomes, healthcare systems and diabetes care providers should move toward routine integration of mental health services in diabetes care settings [17,18,21]. It should become standard practice to have mental health screening (for depression, anxiety, distress) and, importantly, to have resources in place for providing care when patients screen positive. This might involve embedding mental health professionals in diabetes clinics or establishing collaborative care programs. The refrain from our findings is that *mental health integration is not optional but essential for comprehensive diabetes care*.
- **Training and Workforce:** To implement integrated care at scale, there is a need to train more providers in delivering psychosocial care. Primary care physicians and endocrinologists should be trained to recognize and initiate management of common mental health conditions in diabetes (e.g., using motivational interviewing, basic psychopharmacology for antidepressants) and to work in teams with mental health specialists [16,19]. At the same time, more diabetes-knowledgeable mental health professionals (psychologists, clinical social workers) are needed. Task-sharing models can be beneficial: for example, training nurses or community health workers to deliver brief mental health interventions under supervision, which has shown promise in resource-limited settings.
- **Patient Education and Engagement:** Integrating care is not only about providers; patients and families should be educated that emotional well-being is a part of diabetes management. Reducing stigma around seeking mental health support can improve engagement. In practice, when mental healthcare is offered as part of diabetes care, patients may be more receptive (seeing it as routine). Providers should introduce mental health screenings and referrals in a normalizing way—e.g., “We ask all our patients about mood and stress, because it is an important part of diabetes health.” This approach can increase uptake of services, as demonstrated by higher referral completion rates in co-located models.
- **Integrated Care in Different Settings:** For large hospital systems, co-locating behavioral health in diabetes or primary care clinics can be highly effective to facilitate warm hand-offs. Smaller practices might use a collaborative care approach where they partner with off-site mental health providers via telehealth or shared care plans. The use of telemedicine can extend integrated care to rural areas where specialist access is limited [24,27]. Our findings showed telehealth interventions can be effective, but ensuring patients can use the technology is key. Hybrid models (combining in-person and digital) may work best.
- **Policy and Funding:** Sustainable integration requires supportive policies. Fee-for-service models often segregate mental health reimbursement from medical care, which is a barrier. Payers and policymakers should consider blended or collaborative care payment models (for example, the Collaborative Care Model has CPT billing codes in the US). Reimbursement structures need realignment so that team-based care and care coordination activities (often not reimbursed in traditional models) are supported. Additionally, including mental health metrics in diabetes quality dashboards and pay-for-performance programs would incentivize providers and organizations to prioritize this aspect. For instance, tracking the percentage of diabetes patients who receive depression screening and treatment as a quality indicator could drive integration.
- **Addressing Disparities:** Integrated care can improve access to mental health support for vulnerable groups, such as ethnic minorities or low-income patients, who typically face barriers in traditional mental health settings. By bringing services to where patients already receive care (their diabetes clinic), we lower barriers. Our review noted that integrated programs, especially when augmented by telehealth or community health workers, can reduce structural obstacles and improve continuity for underserved populations. Nonetheless, vigilance is required to ensure new models do not inadvertently exclude some groups—for example, digital programs must be made user-friendly and accessible to those with low tech literacy. Culturally tailored approaches, like employing bilingual mental health providers or culturally adapted interventions [23,27] were highlighted as crucial for engagement. Health systems should invest in culturally competent integrated care to maximize benefit across diverse patient populations.

In summary, the practical implication is a call to action for healthcare providers and systems to weave mental health services into the fabric of diabetes care. This includes structural changes (clinic workflows, referral processes, billing), team training, and patient-centered communication. Given the evidence that integrated care can lower complication rates and possibly healthcare costs in the long run, it aligns with both patient well-being and health system sustainability goals.

### 4.4. Limitations of the Evidence Base

While the overall evidence is supportive of integrated care [20,21,22,23], there are important limitations to acknowledge, some of which reflect the limitations of this review and some inherent to the literature:

- **Research Design and Quality:** Not all included studies were high-quality RCTs. We included some quasi-experimental studies to capture real-world implementations, and these have inherent biases. The lack of blinding in many trials could inflate perceived effects on subjective outcomes (like patient-reported depression symptoms) [33,39]. Additionally, a few studies had small sample sizes or short follow-up durations, limiting the robustness of conclusions about long-term effects. As noted earlier, heterogeneity in study design and execution means our findings are based on a diverse set of studies with varying internal validity. We attempted to account for risk of bias in interpreting results, but a formal meta-regression on study quality was not feasible due to the narrative approach.
- **Population Gaps:** There is a notable evidence gap for certain populations. Most research has focused on middle-aged and older adults with type 2 diabetes. We found very few studies specifically targeting children or adolescents with diabetes [21]. Youth with type 1 diabetes, for example, face unique psychosocial challenges and developmental issues; integrated care models might need adaptation for pediatric endocrinology settings. The absence of robust studies in this group is a limitation—our findings may not directly generalize to pediatric diabetes care. Likewise, pregnant women with diabetes (e.g., gestational diabetes or type 1/type 2 in pregnancy) were not explicitly studied in the context of integrated mental healthcare and could benefit from future research.
- **Outcome Measures:** The studies used a variety of outcome measures for mental health (different scales for depression, some focusing on distress, etc.), which makes direct comparison difficult. Few studies examined long-term “hard” outcomes like incidence of diabetes complications or mortality [33,38]. It stands to reason that improved HbA1c and mental health might eventually translate into fewer complications and longer life, but this was beyond the scope of existing research. Also, quality of life, while included in some studies, was not universal; thus, our understanding of how much integrated care improves overall life satisfaction or functioning is incomplete.
- **Follow-up Duration:** Most trials only reported outcomes up to 12 months. As such, we do not fully know if the improvements in depression and glycemic control are sustained over years or if patients might relapse when integrated programs end. Long-term sustainability and whether periodic “boosters” are needed remain unclear. Additionally, any potential long-term adverse effects or patients’ eventual reliance on the integrated support (and outcomes after withdrawal of extra support) are not well studied.
- **Generalizability:** As mentioned, the **geographic distribution** of studies leans heavily toward high-income countries. Caution is warranted in extrapolating to low-resource settings where healthcare infrastructure and workforces differ. The fact that implementation is “patchy” and evidence mostly Western raises the issue of generalizability. Socio-cultural factors play a role in mental health stigma and help-seeking; integrated care that works in one culture may need modification in another. There is a need for more international research to validate these models across different healthcare systems and cultures.
- **Publication and Reporting Bias:** It is possible that studies showing positive results were more likely to be published, while negative or null trials (if any) might be underreported. We did not find clear evidence of unpublished large trials, but given the relatively uniformly positive tone of published studies, one should consider that some smaller efforts that found no improvement may not have made it to publication. This review attempted to be comprehensive, but we cannot rule out some degree of publication bias.
- **This Review’s Limitations:** Finally, with regard to our review process, we synthesized evidence across different study types without performing a formal meta-analysis for every outcome due to heterogeneity. While this allowed us to capture a broad picture, it means the review does not provide a single summary effect size for integrated care’s impact. Also, although we followed systematic methods, certain choices (like inclusion of quasi-experimental studies) and the qualitative nature of some synthesis could introduce subjectivity. We did not quantitatively assess inter-rater agreement for study inclusion or bias assessment (relying on consensus resolution). Nonetheless, we believe these limitations do not invalidate the overall conclusions but suggest that some caution and the need for further research remain.

### 4.5. Comparison with Other Reviews

Our findings are consistent with and extend those of previous reviews. Earlier systematic reviews (e.g., Atlantis et al. 2014 [20], and a 2018 Cochrane [40] review on psychological interventions in diabetes) similarly found that collaborative care improves depression and can slightly improve glycemic control. We included newer studies such as technology-based interventions and recent large trials, which strengthen the evidence base and also highlight innovations (like digital health) that prior reviews did not cover in detail. A recent meta-analysis in *Diabetes Care* (2024) [23] focusing on depression-care integration in diabetes corroborated our results, particularly emphasizing that integrated care is associated with improved glycemic and depression outcomes. Our review goes further by discussing anxiety, distress, and system outcomes, offering a more comprehensive view beyond just depression [24]. 

Another related area is the integration of care for people with serious mental illness who develop diabetes; while that was not the focus of our review (we looked from the diabetes perspective outwards), reviews in that area also support collaborative care models. Thus, whether one starts from the diabetes population or mental health population, the message is similar: integrated, coordinated care yields better outcomes than fragmented care.

### 4.6. Future Research Directions

Our review highlights several avenues for future investigation to address the evidence gaps and open questions:

- **Long-Term and Sustainability Studies:** There is a need for studies that look at outcomes beyond one year. Future RCTs or follow-up studies should examine whether improvements from integrated care are maintained over 2, 3, or 5 years. They should also evaluate if continuous integrated care is necessary or if patients can be stepped down to usual care after improvement without relapse. This ties into questions of the long-term viability and cost-effectiveness of keeping such programs running indefinitely.
- **Focus on Diverse Populations:** Research should focus on pediatrics (children and adolescents) with diabetes—for instance, testing integrated behavioral health interventions in pediatric diabetes clinics or family-based models. Additionally, more trials in low- and middle-income countries are needed [16]. These could test task-shifting approaches (training lay counselors to provide mental healthcare as part of diabetes programs, for example) which might be more feasible in low-resource settings. Culturally tailored integrated care interventions, perhaps employing community health workers or peer supporters, merit testing in diverse ethnic communities to ensure the approach works for all.
- **Specific Mental Health Conditions:** While depression has been extensively studied, anxiety disorders, diabetes distress, and cognitive impairment [30]. Very few studies in diabetes deserve more attention. For example, does integrating care help prevent or slow cognitive decline in older diabetic patients? Can anxiety-focused interventions (like CBT for anxiety) integrate into diabetes visits improve both anxiety and diabetes outcomes? These specific questions remain under-explored.
- **Component Analysis:** Future studies might dismantle which components of integrated care are most critical. Is it the presence of a care manager, the psychiatric case review, the therapy sessions, or simply the systematic monitoring? Trials or implementation studies that vary one component at a time (factorial designs) could help optimize integrated care models for maximum impact at minimum cost.
- **Digital and Hybrid Models:** As technology evolves, research should continue to evaluate digital integrated care tools (apps, telehealth, remote monitoring). Particularly, there should be focus on how to effectively combine digital approaches with human care—for example, using automated mood tracking plus nurse follow-up—and testing those against traditional models. Ensuring equity in digital health (designing for low literacy, different languages, and accessible formats) should be part of these studies so that digital integration does not widen disparities [19].
- **Policy and Systems Research:** Another area is health services research on *implementation*: how to implement integrated care widely. Studying models of care integration in various healthcare systems, identifying barriers (such as those financial or regulatory mentioned) and testing interventions to overcome them (like new payment models, training programs) will be crucial. Essentially, now that efficacy is shown, how do we implement at scale? Research might include pragmatic trials or quality improvement initiatives that also collect outcome data.
- **Cost–Benefit Over Time:** More economic evaluations in diverse settings would bolster the case to policymakers. For example, do integrated programs save costs in a government-funded health system (like the NHS in the UK) as they appear to in some US systems? Long-term cost–benefit analyses factoring in avoided complications could strengthen the argument for funding integrated care [53].

### 4.7. Strengths and Weaknesses of This Review

To contextualize, the strengths of this review include a broad, up-to-date search capturing a range of study designs and integration models, and a thorough qualitative synthesis that covers clinical and system-level outcomes. By structuring the review with PRISMA guidelines and PICO, we have clearly defined the scope and systematically appraised evidence, which should aid clinicians, researchers, and decision-makers in understanding the current state of knowledge.

However, a weakness of this review is the reliance on narrative integration of results due to heterogeneity; we did not provide a single effect size estimate for outcomes like depression or HbA1c (though we cited meta-analyses that did). Also, the inclusion of varied study designs means the evidence quality is mixed—we did not strictly limit to RCTs, which could be seen as a limitation, but it was a deliberate choice to include real-world data. We attempted to mitigate bias via dual screening and extraction, but as with any systematic review, publication bias and the quality of the underlying studies ultimately influence the strength of conclusions.

## 5. Conclusions

This review demonstrates that integrating mental health services into diabetes care consistently improves depression and diabetes-related distress, with modest but clinically meaningful improvements in glycemic control. The integration of mental health services into diabetes care is a vital and evidence-based strategy for improving patient outcomes. This systematic review, encompassing diverse care models from collaborative team-based approaches to co-located clinics and digital interventions, demonstrates that addressing psychological needs within diabetes management leads to better glycemic control, reduced depression and anxiety, improved adherence, and enhanced quality of life for individuals with diabetes. These findings affirm that the historical separation of mental and physical healthcare is no longer tenable for chronic illnesses like diabetes—comprehensive, patient-centered care must treat the whole person.

Despite the positive evidence, implementation in practice remains uneven globally. To bridge this gap, healthcare systems and providers should heed the call to action: mental health should be embedded into diabetes care as a standard of care, not as an afterthought. Practically, this means routine mental health screening in diabetes clinics, ready access to mental health professionals or collaborative care programs for diabetes patients, and healthcare policies that facilitate these practices (through training, funding, and quality incentives). Integrated care models have shown potential not only to improve individual patient outcomes but also to reduce broader healthcare utilization and costs by preventing crises and complications.

Future efforts should focus on sustaining and scaling successful integrated care models, ensuring they are culturally adapted and equitable, and rigorously evaluating their long-term impact on diabetes complications and survival. Research and policy must also invest in closing the treatment gap in regions or populations that lack access to mental health services as part of diabetes care. With diabetes prevalence rising worldwide, the need for holistic care is more pressing than ever. By integrating mental health into diabetes treatment paradigms, we can move closer to truly comprehensive care—care that not only extends life but improves the quality of life for people with diabetes. The evidence to date strongly supports that such integration is both feasible and beneficial. It is time for the healthcare community to translate this evidence into everyday practice, ensuring that no person with diabetes is left to manage the psychological burden of the disease on their own.

Our synthesis highlights three original insights: (i) patients with poorly controlled diabetes (baseline HbA1c > 9%) benefit disproportionately from integrated care, with reductions ≥ 1% in some cohorts; (ii) collaborative and co-located care models are particularly effective at improving treatment adherence and self-management; and (iii) important research gaps remain in pediatric populations, low-income countries, and culturally diverse communities, where tailored models are urgently needed.

**Recommendations:** Health organizations and diabetes clinics should develop and implement integrated care pathways that include mental health evaluation and intervention [54]. Clinicians are encouraged to collaborate across disciplines, and guidelines should continue to emphasize mental health as part of diabetes standards of care. Policymakers should consider funding mechanisms and workforce initiatives to support widespread adoption of integrated care. Ultimately, making mental health integration routine in diabetes care will help close the persistent treatment gap and improve outcomes, fulfilling the promise of truly patient-centered care for the millions affected by this challenging chronic condition.

## Figures and Tables

**Table 2:** Summary of Outcomes Improved by Integrated Mental Healthcare in Diabetes.

Domain	Effect	Evidence Highlights
**Depression**	↓ symptoms, ↑ remission	8 RCT meta-analysis [20] COMPASS 2016 [26]
**HbA1c**	↓ modest (−0.3% to −0.5%)	TEAMcare, Atlantis meta-analysis [20]
**Anxiety/Distress**	↓ scores	[21,23]
**Adherence**	↑ medication & SMBG	Several RCTs + meta-analysis
**QOL**	Mild ↑	SF-36 and patient satisfaction
**Costs**	Cost-effective	[21]

## Data Availability

No new data were created or analyzed in this study. Data sharing is not applicable to this article.
